# Poly[μ-aqua-diaqua(μ_3_-1*H*-benzimid­azole-5-carboxylato-κ^3^ *N* ^3^:*O*,*O*′)(μ_2_-1*H*-benzimidazole-5-carboxylato-κ^3^ *N* ^3^:*O*:*O*′)-μ_5_-sulfato-μ_4_-sulfato-tri­cadmium]

**DOI:** 10.1107/S1600536811034477

**Published:** 2011-08-27

**Authors:** Lai-Chen Chen, Shu-Min Huo, Hao-Zhao Chen, Ya-Qing Yang, Rong-Hua Zeng

**Affiliations:** a School of Chemistry and Environment, South China Normal University, Guangzhou 510006, People’s Republic of China; b Key Laboratory of Technology of Electrochemical Energy Storage and Power Generation in Guangdong Universities, South China Normal University, Guangzhou 510006, People’s Republic of China

## Abstract

The asymmetric unit of the title compound, [Cd_3_(C_8_H_5_N_2_O_2_)_2_(SO_4_)_2_(H_2_O)_3_]_*n*_, contains three Cd^II^ ions, two sulfate anions, two 1*H*-benzimidazole-5-carboxyl­ate (H_2_bic) ligands and three coordinated water mol­ecules. One Cd^II^ ion is six-coordinated and exhibits a distorted octa­hedral geometry, while the other two Cd^II^ ions are seven-coordinated, displaying a distorted penta­gonal–bipyramidal geometry. The Cd^II^ ions are bridged by two types of sulfate anions, producing inorganic chains along [100]. These chains are further connected by the H_2_bic ligands, leading to a three-dimensional framework. N—H⋯O and O—H⋯O hydrogen bonds and π–π inter­actions between the imidazole and benzene rings [centroid–centroid distances = 3.953 (2), 3.507 (2), 3.407 (2) and 3.561 (2) Å] further stabilize the crystal structure.

## Related literature

For background to 1*H*-benzimidazole-5-carboxyl­ate com­plexes, see: Gao *et al.* (2011 ▶); Guo *et al.* (2007 ▶); Peng, Ma *et al.* (2010 ▶); Peng, Qiu *et al.* (2010 ▶); Yao *et al.* (2008 ▶). 
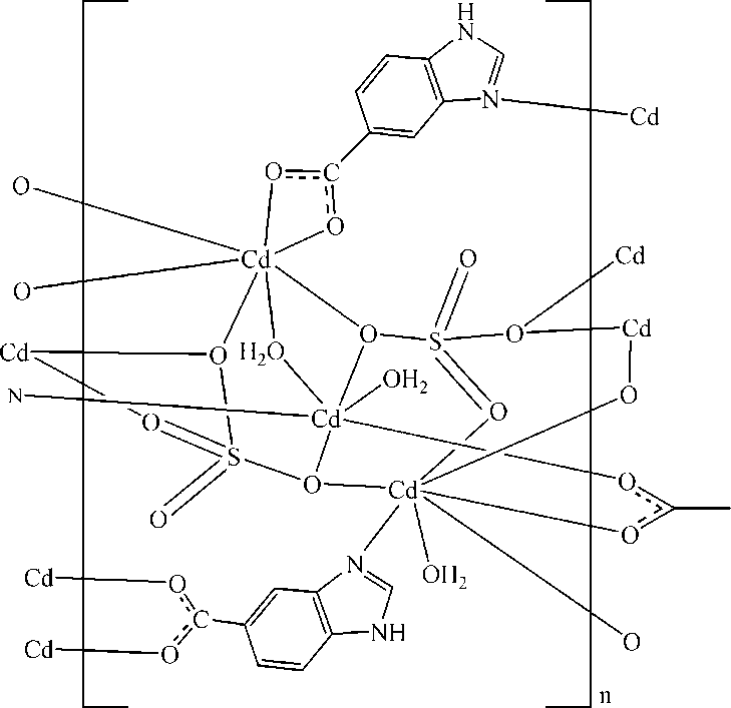


## Experimental

### 

#### Crystal data

[Cd_3_(C_8_H_5_N_2_O_2_)_2_(SO_4_)_2_(H_2_O)_3_]
*M* *_r_* = 905.65
Triclinic, 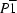

*a* = 6.5932 (8) Å
*b* = 13.0463 (16) Å
*c* = 13.5933 (16) Å
α = 104.313 (1)°
β = 96.662 (1)°
γ = 97.646 (1)°
*V* = 1109.3 (2) Å^3^
*Z* = 2
Mo *K*α radiation
μ = 3.13 mm^−1^
*T* = 298 K
0.30 × 0.27 × 0.25 mm

#### Data collection

Bruker APEXII CCD diffractometer
Absorption correction: multi-scan (*SADABS*; Bruker, 2001 ▶) *T*_min_ = 0.454, *T*_max_ = 0.508
5772 measured reflections
3935 independent reflections
3593 reflections with *I* > 2σ(*I*)
*R*_int_ = 0.020

#### Refinement

*R*[*F* ^2^ > 2σ(*F* ^2^)] = 0.025
*wR*(*F* ^2^) = 0.067
*S* = 1.06
3935 reflections
361 parameters
1 restraint
H-atom parameters constrained
Δρ_max_ = 0.56 e Å^−3^
Δρ_min_ = −0.71 e Å^−3^

### 

Data collection: *APEX2* (Bruker, 2007 ▶); cell refinement: *SAINT* (Bruker, 2007 ▶); data reduction: *SAINT*; program(s) used to solve structure: *SHELXS97* (Sheldrick, 2008 ▶); program(s) used to refine structure: *SHELXL97* (Sheldrick, 2008 ▶); molecular graphics: *ORTEPIII* (Burnett & Johnson, 1996 ▶) and *PLATON* (Spek, 2009 ▶); software used to prepare material for publication: *SHELXL97*.

## Supplementary Material

Crystal structure: contains datablock(s) I, global. DOI: 10.1107/S1600536811034477/hy2460sup1.cif

Structure factors: contains datablock(s) I. DOI: 10.1107/S1600536811034477/hy2460Isup2.hkl

Additional supplementary materials:  crystallographic information; 3D view; checkCIF report

## Figures and Tables

**Table 1:** Hydrogen-bond geometry (Å, °)

*D*—H⋯*A*	*D*—H	H⋯*A*	*D*⋯*A*	*D*—H⋯*A*
N2—H2⋯O10^i^	0.86	1.98	2.836 (4)	176
N4—H4*A*⋯O7^ii^	0.86	1.98	2.716 (4)	143
O1*W*—H1*W*⋯O3^iii^	0.85	1.91	2.736 (4)	163
O1*W*—H2*W*⋯O2^iv^	0.85	1.91	2.734 (4)	165
O2*W*—H3*W*⋯O3^v^	0.85	1.99	2.770 (4)	153
O2*W*—H4*W*⋯O10^v^	0.85	2.01	2.687 (4)	136
O3*W*—H5*W*⋯O8^i^	0.85	2.23	2.925 (4)	139
O3*W*—H6*W*⋯O4^vi^	0.85	2.09	2.918 (4)	166

Symmetry codes: (i) 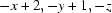; (ii) 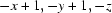; (iii) 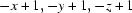; (iv) 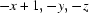; (v) 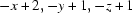; (vi) 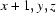.

**Fig. 1.**
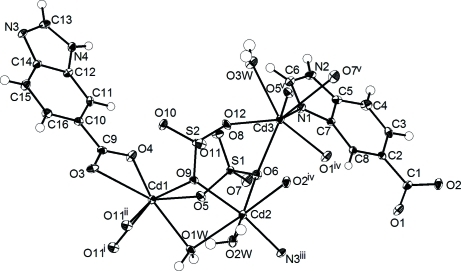
The asymmetric unit of the title compound, showing displacement ellipsoids drawn at the 30% probability level. [Symmetry codes: (i) x-1, y, z; (ii) -x+2, -y+1, -z+1; (iii) x, y-1, z; (iv) -x+2, -y, -z; (v) x+1, y, z.]

**Fig. 2.**
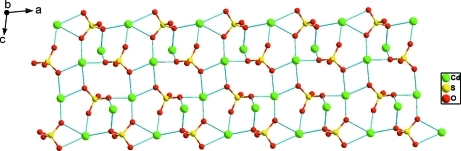
The one-dimensional chain extending along [1 0 0], formed by Cd atoms and sulfate anions.

**Fig. 3.**
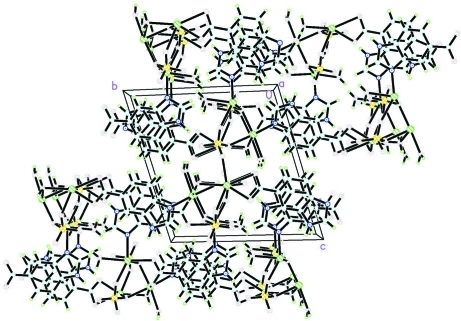
A view of the three-dimensional structure of the title compound.

## supplementary crystallographic information

### Comment

There is currently much interest in employing N-heterocyclic carboxylic acids as multidentate ligands to design metal coordination polymers. This is because they have versatile coordination modes and can form high-dimensional polymers through hydrogen-bonding interactions in the process of self-assembly. 1*H*-Benzimidazole-5-carboxylic acid (H_2_bic), having two N atoms of an aromatic group and one carboxylate group, is a good candidate for preparing novel coordination polymers. Up to now, one-, two- and three-dimensional coordination polymers constructed from the H_2_bic ligand have been reported (Gao *et al.*, 2011; Guo *et al.*, 2007; Peng *et al.*, 2010a,b; Yao *et al.*, 2008). Herein we report the synthesis and crystal structure of the title complex.

As is shown in Fig. 1, the asymmetric unit of the title compound consists of three crystallographically independent Cd^II^ ions, two SO_4_^2-^ anions, two H_2_bic ligands and three coordinated water molecules. The Cd2 atom is six-coordinated by one O atom and one N atom from two different H_2_bic ligands, two O atoms from two SO_4_^2-^ anions and two water molecules, forming a distorted octahedral geometry. Both Cd1 and Cd3 atoms are seven-coordinated, displaying a distorted pentagonal-bipyramidal geometry. The Cd1 atom is coordinated by two O atoms from one H_2_bic ligand, four O atoms from four SO_4_^2-^ anions and one water molecule, while Cd3 atom is surrounded by one O atom and one N atom from two H_2_bic ligands, four O atoms from three SO_4_^2-^ anions and one water molecule. The Cd—O bond lengths range from 2.266 (3) to 2.532 (3) Å and the Cd—N distances vary from 2.230 (3) to 2.272 (3) Å.

In the title compound, the SO_4_^2-^ anions adopt two coordination modes. One is a µ_4_-mode, bridging four Cd^II^ ions and the other is a µ_5_-mode, bridging five Cd^II^ ions. As is described in Fig. 2, the Cd^II^ ions are bridged by the two types of SO_4_^2-^ anions, producing a one-dimensional inorganic chain along [1 0 0]. These chains are further bridged by the carboxylate and imidazole groups of the H_2_bic ligands, resulting in a three-dimensional framework (Fig. 3). To the best of our knowledge, the title compound is the first three-dimensional transition metal coordination polymer based on H_2_bic ligand. N—H···O and O—H···O hydrogen bonds (Table 1) and π–π interactions between the imidazole and benzene rings [centroid–centroid distances = 3.953 (2), 3.507 (2), 3.407 (2) and 3.561 (2) Å] further stabilize the crystal structure.

### Experimental

A mixture of CdSO_4_ (0.208 g, 1 mmol), H_2_bic (0.162 g, 1 mmol) and water (10 ml) was stirred vigorously for 30 min and then sealed in a 20 ml Teflon-lined stainless-steel autoclave. The autoclave was heated and maintained at 433 K for 3 days, and then cooled to room temperature at 5 K h^-1^. Colorless block crystals were obtained.

### Refinement

H atoms of the H_2_bic ligands were positioned geometrically and refined as riding atoms, with C—H = 0.93 and N—H = 0.86 Å, and with *U*_iso_(H) = 1.2*U*_eq_(C,N). The water H atoms were located in a difference Fourier map and refined as riding, with a distance restraint of O—H = 0.85 Å and with *U*_iso_(H) = 1.2*U*_eq_(O).

### Crystal data

**Table:**

[Cd_3_(C_8_H_5_N_2_O_2_)_2_(SO_4_)_2_(H_2_O)_3_]	*Z* = 2
*M**_r_* = 905.65	*F*(000) = 872
Triclinic, *P*1	*D*_x_ = 2.711 Mg m^−^^3^
Hall symbol: -P 1	Mo *K*α radiation, λ = 0.71073 Å
*a* = 6.5932 (8) Å	Cell parameters from 3917 reflections
*b* = 13.0463 (16) Å	θ = 2.5–25.2°
*c* = 13.5933 (16) Å	µ = 3.13 mm^−^^1^
α = 104.313 (1)°	*T* = 298 K
β = 96.662 (1)°	Block, colorless
γ = 97.646 (1)°	0.30 × 0.27 × 0.25 mm
*V* = 1109.3 (2) Å^3^	

### Data collection

**Table:**

Bruker APEXII CCD diffractometer	3935 independent reflections
Radiation source: fine-focus sealed tube	3593 reflections with *I* > 2σ(*I*)
graphite	*R*_int_ = 0.020
φ and ω scans	θ_max_ = 25.2°, θ_min_ = 1.9°
Absorption correction: multi-scan (*SADABS*; Bruker, 2001)	*h* = −7→7
*T*_min_ = 0.454, *T*_max_ = 0.508	*k* = −13→15
5772 measured reflections	*l* = −16→13

### Refinement

**Table:**

Refinement on *F*^2^	Primary atom site location: structure-invariant direct methods
Least-squares matrix: full	Secondary atom site location: difference Fourier map
*R*[*F*^2^ > 2σ(*F*^2^)] = 0.025	Hydrogen site location: inferred from neighbouring sites
*wR*(*F*^2^) = 0.067	H-atom parameters constrained
*S* = 1.06	*w* = 1/[σ^2^(*F*_o_^2^) + (0.0305*P*)^2^ + 1.2679*P*] where *P* = (*F*_o_^2^ + 2*F*_c_^2^)/3
3935 reflections	(Δ/σ)_max_ = 0.001
361 parameters	Δρ_max_ = 0.56 e Å^−^^3^
1 restraint	Δρ_min_ = −0.71 e Å^−^^3^

### Fractional atomic coordinates and isotropic or equivalent isotropic displacement parameters (Å^2^)

**Table:**

	*x*	*y*	*z*	*U*_iso_*/*U*_eq_	
Cd1	0.61304 (4)	0.48371 (2)	0.37140 (2)	0.01777 (9)	
Cd2	0.84093 (4)	0.24373 (2)	0.30872 (2)	0.01851 (9)	
Cd3	1.17453 (4)	0.31863 (2)	0.10959 (2)	0.01829 (9)	
S1	0.66513 (14)	0.33480 (8)	0.11673 (7)	0.0152 (2)	
S2	1.12894 (14)	0.49174 (7)	0.36347 (7)	0.0135 (2)	
N1	1.0207 (5)	0.2733 (3)	−0.0571 (2)	0.0197 (7)	
C6	1.0162 (6)	0.3440 (3)	−0.1108 (3)	0.0225 (9)	
H6	1.0520	0.4177	−0.0821	0.027*	
C8	0.9189 (6)	0.0704 (3)	−0.1153 (3)	0.0154 (8)	
H8	0.9361	0.0589	−0.0502	0.018*	
C7	0.9513 (6)	0.1732 (3)	−0.1279 (3)	0.0156 (8)	
C3	0.8338 (6)	0.0033 (3)	−0.3013 (3)	0.0218 (9)	
H3	0.8002	−0.0553	−0.3589	0.026*	
C4	0.8563 (7)	0.1046 (4)	−0.3146 (3)	0.0246 (9)	
H4	0.8338	0.1158	−0.3797	0.030*	
C5	0.9148 (6)	0.1906 (3)	−0.2263 (3)	0.0191 (8)	
C2	0.8598 (6)	−0.0150 (3)	−0.2030 (3)	0.0161 (8)	
C16	0.6557 (7)	0.8881 (3)	0.4017 (3)	0.0226 (9)	
H16	0.6714	0.8841	0.4695	0.027*	
C9	0.6064 (6)	0.6877 (3)	0.3474 (3)	0.0195 (9)	
C10	0.6106 (6)	0.7927 (3)	0.3226 (3)	0.0183 (8)	
C15	0.6775 (7)	0.9878 (3)	0.3828 (3)	0.0240 (9)	
H15	0.7064	1.0506	0.4361	0.029*	
O6	0.8233 (4)	0.2704 (2)	0.1470 (2)	0.0239 (6)	
O5	0.5458 (5)	0.3615 (2)	0.2041 (2)	0.0259 (7)	
O8	0.7711 (5)	0.4300 (2)	0.0964 (2)	0.0303 (7)	
O4	0.5783 (5)	0.6023 (2)	0.2762 (2)	0.0275 (7)	
O3	0.6395 (5)	0.6846 (2)	0.4412 (2)	0.0257 (6)	
O9	0.9346 (4)	0.4272 (2)	0.3769 (2)	0.0198 (6)	
O10	1.0948 (5)	0.6016 (2)	0.3744 (2)	0.0238 (6)	
O12	1.1865 (5)	0.4465 (2)	0.2640 (2)	0.0276 (7)	
O7	0.5172 (5)	0.2693 (2)	0.0280 (2)	0.0295 (7)	
C11	0.5815 (6)	0.7939 (3)	0.2201 (3)	0.0182 (8)	
H11	0.5493	0.7311	0.1668	0.022*	
C12	0.6033 (6)	0.8950 (3)	0.2021 (3)	0.0170 (8)	
C14	0.6545 (6)	0.9901 (3)	0.2807 (3)	0.0190 (8)	
O11	1.2984 (4)	0.4879 (2)	0.4437 (2)	0.0231 (6)	
N2	0.9543 (5)	0.2985 (3)	−0.2121 (3)	0.0234 (8)	
H2	0.9420	0.3318	−0.2591	0.028*	
N4	0.5920 (5)	0.9255 (3)	0.1113 (2)	0.0188 (7)	
H4A	0.5611	0.8834	0.0501	0.023*	
C1	0.8336 (6)	−0.1274 (3)	−0.1935 (3)	0.0188 (9)	
O1	0.8145 (5)	−0.1439 (2)	−0.1085 (2)	0.0266 (7)	
N3	0.6749 (5)	1.0765 (3)	0.2371 (2)	0.0191 (7)	
C13	0.6382 (6)	1.0324 (3)	0.1363 (3)	0.0222 (9)	
H13	0.6441	1.0719	0.0881	0.027*	
O2	0.8337 (4)	−0.2035 (2)	−0.2739 (2)	0.0245 (7)	
O2W	0.9522 (5)	0.2349 (2)	0.4699 (2)	0.0254 (7)	
H3W	1.0790	0.2374	0.4926	0.030*	
H4W	0.8920	0.2573	0.5212	0.030*	
O3W	1.3093 (5)	0.4879 (2)	0.0835 (2)	0.0305 (7)	
H5W	1.3544	0.5197	0.0409	0.037*	
H6W	1.3685	0.5238	0.1434	0.037*	
O1W	0.5431 (4)	0.3032 (2)	0.3864 (2)	0.0199 (6)	
H1W	0.5114	0.3053	0.4457	0.024*	
H2W	0.4386	0.2693	0.3422	0.024*	

### Atomic displacement parameters (Å^2^)

**Table:**

	*U*^11^	*U*^22^	*U*^33^	*U*^12^	*U*^13^	*U*^23^
Cd1	0.01897 (17)	0.01555 (16)	0.02043 (16)	0.00466 (12)	0.00520 (12)	0.00592 (12)
Cd2	0.02124 (17)	0.01475 (16)	0.01756 (16)	0.00124 (12)	0.00324 (12)	0.00147 (12)
Cd3	0.02241 (17)	0.01492 (16)	0.01583 (16)	0.00122 (12)	0.00240 (12)	0.00221 (12)
S1	0.0164 (5)	0.0140 (5)	0.0139 (5)	0.0007 (4)	0.0021 (4)	0.0026 (4)
S2	0.0122 (5)	0.0131 (5)	0.0142 (5)	0.0008 (4)	0.0014 (4)	0.0027 (4)
N1	0.0241 (19)	0.0117 (17)	0.0215 (18)	0.0023 (14)	0.0020 (14)	0.0019 (14)
C6	0.025 (2)	0.017 (2)	0.025 (2)	0.0022 (17)	0.0028 (17)	0.0066 (17)
C8	0.020 (2)	0.0149 (19)	0.0127 (18)	0.0044 (16)	0.0034 (15)	0.0046 (15)
C7	0.0140 (19)	0.016 (2)	0.0164 (19)	0.0009 (15)	0.0028 (15)	0.0039 (16)
C3	0.025 (2)	0.025 (2)	0.013 (2)	0.0055 (18)	0.0035 (16)	−0.0011 (17)
C4	0.028 (2)	0.030 (2)	0.016 (2)	0.0043 (19)	−0.0018 (17)	0.0100 (18)
C5	0.014 (2)	0.023 (2)	0.023 (2)	0.0033 (16)	0.0021 (16)	0.0121 (18)
C2	0.0133 (19)	0.017 (2)	0.019 (2)	0.0032 (15)	0.0044 (15)	0.0044 (16)
C16	0.028 (2)	0.024 (2)	0.015 (2)	0.0016 (18)	0.0019 (17)	0.0057 (17)
C9	0.012 (2)	0.022 (2)	0.028 (2)	0.0039 (16)	0.0050 (16)	0.0122 (18)
C10	0.016 (2)	0.020 (2)	0.020 (2)	0.0022 (16)	0.0036 (16)	0.0070 (17)
C15	0.029 (2)	0.018 (2)	0.021 (2)	0.0003 (18)	−0.0004 (18)	0.0010 (17)
O6	0.0229 (16)	0.0247 (16)	0.0279 (16)	0.0078 (13)	0.0035 (12)	0.0120 (13)
O5	0.0269 (17)	0.0300 (17)	0.0221 (15)	0.0058 (13)	0.0104 (12)	0.0059 (13)
O8	0.0300 (18)	0.0241 (17)	0.0386 (18)	−0.0016 (14)	0.0098 (14)	0.0131 (14)
O4	0.0368 (18)	0.0162 (16)	0.0282 (16)	0.0029 (13)	−0.0001 (13)	0.0069 (13)
O3	0.0304 (17)	0.0252 (13)	0.0252 (16)	0.0019 (13)	0.0018 (13)	0.0162 (12)
O9	0.0146 (14)	0.0201 (15)	0.0229 (15)	0.0000 (11)	0.0066 (11)	0.0023 (12)
O10	0.0342 (17)	0.0156 (15)	0.0225 (15)	0.0078 (13)	0.0019 (12)	0.0056 (12)
O12	0.0308 (17)	0.0274 (17)	0.0192 (15)	−0.0008 (13)	0.0144 (13)	−0.0055 (13)
O7	0.0321 (18)	0.0283 (17)	0.0203 (15)	−0.0002 (14)	−0.0061 (13)	−0.0002 (13)
C11	0.020 (2)	0.016 (2)	0.0157 (19)	0.0039 (16)	0.0024 (15)	−0.0004 (16)
C12	0.016 (2)	0.015 (2)	0.019 (2)	0.0006 (16)	−0.0027 (15)	0.0063 (16)
C14	0.018 (2)	0.015 (2)	0.024 (2)	0.0021 (16)	0.0027 (16)	0.0072 (17)
O11	0.0161 (14)	0.0306 (17)	0.0241 (15)	0.0053 (12)	−0.0008 (12)	0.0112 (13)
N2	0.030 (2)	0.0241 (19)	0.0231 (19)	0.0060 (16)	0.0047 (15)	0.0174 (16)
N4	0.0263 (19)	0.0163 (17)	0.0121 (16)	0.0033 (14)	0.0012 (13)	0.0018 (13)
C1	0.012 (2)	0.016 (2)	0.025 (2)	−0.0006 (15)	−0.0027 (16)	0.0043 (17)
O1	0.0368 (18)	0.0158 (15)	0.0251 (16)	0.0014 (13)	−0.0025 (13)	0.0067 (12)
N3	0.0206 (18)	0.0134 (17)	0.0201 (17)	−0.0015 (14)	−0.0009 (14)	0.0030 (14)
C13	0.025 (2)	0.022 (2)	0.021 (2)	0.0019 (18)	−0.0012 (17)	0.0124 (18)
O2	0.0189 (15)	0.0184 (15)	0.0302 (16)	0.0021 (12)	0.0015 (12)	−0.0032 (13)
O2W	0.0279 (17)	0.0284 (17)	0.0182 (14)	0.0057 (13)	0.0020 (12)	0.0035 (13)
O3W	0.0418 (19)	0.0237 (16)	0.0221 (16)	−0.0005 (14)	−0.0026 (13)	0.0057 (13)
O1W	0.0180 (15)	0.0215 (15)	0.0164 (14)	−0.0008 (12)	0.0041 (11)	−0.0001 (11)

### Geometric parameters (Å, °)

**Table:**

Cd1—O4	2.266 (3)	C3—C4	1.368 (6)
Cd1—O9	2.335 (3)	C3—C2	1.410 (5)
Cd1—O5	2.386 (3)	C3—H3	0.9300
Cd1—O11^i^	2.398 (3)	C4—C5	1.401 (6)
Cd1—O1W	2.403 (3)	C4—H4	0.9300
Cd1—O11^ii^	2.437 (3)	C5—N2	1.358 (5)
Cd1—O3	2.532 (3)	C2—C1	1.494 (5)
Cd2—N3^iii^	2.229 (3)	C16—C15	1.380 (6)
Cd2—O2W	2.264 (3)	C16—C10	1.399 (6)
Cd2—O6	2.301 (3)	C16—H16	0.9300
Cd2—O9	2.312 (3)	C9—O4	1.259 (5)
Cd2—O2^iv^	2.349 (3)	C9—O3	1.280 (5)
Cd2—O1W	2.461 (3)	C9—C10	1.487 (5)
Cd3—N1	2.271 (3)	C10—C11	1.390 (5)
Cd3—O1^iv^	2.287 (3)	C15—C14	1.388 (6)
Cd3—O12	2.321 (3)	C15—H15	0.9300
Cd3—O3W	2.394 (3)	C11—C12	1.392 (5)
Cd3—O6	2.462 (3)	C11—H11	0.9300
Cd3—O5^v^	2.551 (3)	C12—N4	1.384 (5)
S1—O8	1.445 (3)	C12—C14	1.394 (6)
S1—O7	1.460 (3)	C14—N3	1.396 (5)
S1—O5	1.495 (3)	N2—H2	0.8600
S1—O6	1.506 (3)	N4—C13	1.335 (5)
S2—O12	1.451 (3)	N4—H4A	0.8600
S2—O10	1.454 (3)	C1—O1	1.243 (5)
S2—O11	1.483 (3)	C1—O2	1.282 (5)
S2—O9	1.493 (3)	N3—C13	1.328 (5)
N1—C6	1.312 (5)	C13—H13	0.9300
N1—C7	1.399 (5)	O2W—H3W	0.8500
C6—N2	1.347 (5)	O2W—H4W	0.8500
C6—H6	0.9300	O3W—H5W	0.8500
C8—C7	1.386 (5)	O3W—H6W	0.8501
C8—C2	1.391 (5)	O1W—H1W	0.8501
C8—H8	0.9300	O1W—H2W	0.8500
C7—C5	1.410 (5)		
			
O4—Cd1—O9	113.71 (10)	C4—C3—H3	119.0
O4—Cd1—O5	80.91 (10)	C2—C3—H3	119.0
O9—Cd1—O5	83.30 (10)	C3—C4—C5	117.3 (4)
O4—Cd1—O11^i^	99.81 (10)	C3—C4—H4	121.3
O9—Cd1—O11^i^	145.80 (9)	C5—C4—H4	121.3
O5—Cd1—O11^i^	109.59 (10)	N2—C5—C4	132.6 (4)
O4—Cd1—O1W	149.15 (10)	N2—C5—C7	106.2 (3)
O9—Cd1—O1W	75.24 (9)	C4—C5—C7	121.2 (4)
O5—Cd1—O1W	70.61 (10)	C8—C2—C3	120.7 (4)
O11^i^—Cd1—O1W	79.48 (9)	C8—C2—C1	119.8 (3)
O4—Cd1—O11^ii^	130.86 (10)	C3—C2—C1	119.5 (3)
O9—Cd1—O11^ii^	80.54 (9)	C15—C16—C10	122.4 (4)
O5—Cd1—O11^ii^	148.10 (10)	C15—C16—H16	118.8
O11^i^—Cd1—O11^ii^	72.16 (10)	C10—C16—H16	118.8
O1W—Cd1—O11^ii^	78.73 (9)	O4—C9—O3	120.0 (4)
O4—Cd1—O3	54.20 (9)	O4—C9—C10	120.0 (4)
O9—Cd1—O3	113.17 (10)	O3—C9—C10	119.9 (4)
O5—Cd1—O3	135.11 (9)	C11—C10—C16	121.2 (4)
O11^i^—Cd1—O3	80.54 (10)	C11—C10—C9	118.7 (4)
O1W—Cd1—O3	152.12 (9)	C16—C10—C9	120.0 (3)
O11^ii^—Cd1—O3	76.75 (9)	C16—C15—C14	116.9 (4)
N3^iii^—Cd2—O2W	101.12 (11)	C16—C15—H15	121.5
N3^iii^—Cd2—O6	88.53 (11)	C14—C15—H15	121.5
O2W—Cd2—O6	164.12 (11)	S1—O6—Cd2	117.49 (16)
N3^iii^—Cd2—O9	166.27 (11)	S1—O6—Cd3	116.06 (15)
O2W—Cd2—O9	84.74 (10)	Cd2—O6—Cd3	110.03 (11)
O6—Cd2—O9	88.70 (10)	S1—O5—Cd1	135.31 (18)
N3^iii^—Cd2—O2^iv^	94.34 (11)	S1—O5—Cd3^i^	101.40 (14)
O2W—Cd2—O2^iv^	85.19 (10)	Cd1—O5—Cd3^i^	117.53 (11)
O6—Cd2—O2^iv^	81.50 (10)	C9—O4—Cd1	99.4 (2)
O9—Cd2—O2^iv^	98.54 (10)	C9—O3—Cd1	86.4 (2)
N3^iii^—Cd2—O1W	93.74 (11)	S2—O9—Cd2	124.43 (15)
O2W—Cd2—O1W	82.80 (10)	S2—O9—Cd1	124.10 (16)
O6—Cd2—O1W	109.37 (9)	Cd2—O9—Cd1	101.86 (10)
O9—Cd2—O1W	74.54 (9)	S2—O12—Cd3	156.42 (19)
O2^iv^—Cd2—O1W	166.62 (10)	C10—C11—C12	115.8 (4)
N1—Cd3—O1^iv^	92.82 (11)	C10—C11—H11	122.1
N1—Cd3—O12	140.73 (11)	C12—C11—H11	122.1
O1^iv^—Cd3—O12	119.93 (11)	N4—C12—C11	131.1 (4)
N1—Cd3—O3W	86.41 (11)	N4—C12—C14	105.8 (3)
O1^iv^—Cd3—O3W	153.63 (11)	C11—C12—C14	123.1 (4)
O12—Cd3—O3W	73.50 (10)	C15—C14—C12	120.5 (4)
N1—Cd3—O6	85.14 (11)	C15—C14—N3	130.6 (4)
O1^iv^—Cd3—O6	81.41 (10)	C12—C14—N3	108.9 (3)
O12—Cd3—O6	79.61 (10)	S2—O11—Cd1^v^	108.68 (15)
O3W—Cd3—O6	124.68 (10)	S2—O11—Cd1^ii^	142.21 (17)
N1—Cd3—O5^v^	135.91 (11)	Cd1^v^—O11—Cd1^ii^	107.84 (10)
O1^iv^—Cd3—O5^v^	86.67 (10)	C6—N2—C5	107.6 (3)
O12—Cd3—O5^v^	71.83 (10)	C6—N2—H2	126.2
O3W—Cd3—O5^v^	75.92 (10)	C5—N2—H2	126.2
O6—Cd3—O5^v^	137.90 (9)	C13—N4—C12	107.3 (3)
O8—S1—O7	112.03 (19)	C13—N4—H4A	126.4
O8—S1—O5	112.00 (18)	C12—N4—H4A	126.4
O7—S1—O5	106.71 (18)	O1—C1—O2	122.5 (4)
O8—S1—O6	108.93 (18)	O1—C1—C2	119.3 (3)
O7—S1—O6	110.12 (18)	O2—C1—C2	118.2 (4)
O5—S1—O6	106.92 (16)	C1—O1—Cd3^iv^	112.1 (2)
O12—S2—O10	111.20 (18)	C13—N3—C14	104.9 (3)
O12—S2—O11	107.91 (18)	C13—N3—Cd2^vi^	123.2 (3)
O10—S2—O11	110.38 (17)	C14—N3—Cd2^vi^	128.5 (3)
O12—S2—O9	110.49 (17)	N3—C13—N4	113.1 (3)
O10—S2—O9	108.24 (17)	N3—C13—H13	123.4
O11—S2—O9	108.60 (16)	N4—C13—H13	123.4
C6—N1—C7	105.7 (3)	C1—O2—Cd2^iv^	116.4 (2)
C6—N1—Cd3	122.0 (3)	Cd2—O2W—H3W	123.0
C7—N1—Cd3	131.4 (2)	Cd2—O2W—H4W	123.8
N1—C6—N2	112.8 (4)	H3W—O2W—H4W	107.7
N1—C6—H6	123.6	Cd3—O3W—H5W	145.3
N2—C6—H6	123.6	Cd3—O3W—H6W	102.8
C7—C8—C2	117.9 (3)	H5W—O3W—H6W	107.7
C7—C8—H8	121.1	Cd1—O1W—Cd2	95.76 (9)
C2—C8—H8	121.1	Cd1—O1W—H1W	108.5
C8—C7—N1	131.6 (3)	Cd2—O1W—H1W	130.5
C8—C7—C5	120.8 (4)	Cd1—O1W—H2W	108.6
N1—C7—C5	107.6 (3)	Cd2—O1W—H2W	104.1
C4—C3—C2	122.0 (4)	H1W—O1W—H2W	107.7
			
O1^iv^—Cd3—N1—C6	−163.5 (3)	O4—Cd1—O3—C9	−0.5 (2)
O12—Cd3—N1—C6	48.4 (4)	O9—Cd1—O3—C9	102.9 (2)
O3W—Cd3—N1—C6	−9.9 (3)	O5—Cd1—O3—C9	−1.6 (3)
O6—Cd3—N1—C6	115.4 (3)	O11^i^—Cd1—O3—C9	−109.9 (2)
O5^v^—Cd3—N1—C6	−75.3 (4)	O1W—Cd1—O3—C9	−154.6 (2)
O1^iv^—Cd3—N1—C7	3.3 (3)	O11^ii^—Cd1—O3—C9	176.3 (2)
O12—Cd3—N1—C7	−144.9 (3)	O12—S2—O9—Cd2	32.9 (2)
O3W—Cd3—N1—C7	156.9 (4)	O10—S2—O9—Cd2	154.85 (17)
O6—Cd3—N1—C7	−77.9 (3)	O11—S2—O9—Cd2	−85.3 (2)
O5^v^—Cd3—N1—C7	91.4 (4)	O12—S2—O9—Cd1	−107.1 (2)
C7—N1—C6—N2	−1.6 (5)	O10—S2—O9—Cd1	14.9 (2)
Cd3—N1—C6—N2	168.1 (3)	O11—S2—O9—Cd1	134.77 (18)
C2—C8—C7—N1	−176.2 (4)	N3^iii^—Cd2—O9—S2	−142.1 (4)
C2—C8—C7—C5	3.3 (6)	O2W—Cd2—O9—S2	101.78 (19)
C6—N1—C7—C8	−178.1 (4)	O6—Cd2—O9—S2	−63.74 (19)
Cd3—N1—C7—C8	13.5 (6)	O2^iv^—Cd2—O9—S2	17.5 (2)
C6—N1—C7—C5	2.4 (4)	O1W—Cd2—O9—S2	−174.3 (2)
Cd3—N1—C7—C5	−166.0 (3)	N3^iii^—Cd2—O9—Cd1	4.9 (5)
C2—C3—C4—C5	2.4 (6)	O2W—Cd2—O9—Cd1	−111.23 (11)
C3—C4—C5—N2	178.4 (4)	O6—Cd2—O9—Cd1	83.25 (11)
C3—C4—C5—C7	0.8 (6)	O2^iv^—Cd2—O9—Cd1	164.46 (10)
C8—C7—C5—N2	178.1 (3)	O1W—Cd2—O9—Cd1	−27.26 (9)
N1—C7—C5—N2	−2.3 (4)	O4—Cd1—O9—S2	26.4 (2)
C8—C7—C5—C4	−3.7 (6)	O5—Cd1—O9—S2	103.35 (19)
N1—C7—C5—C4	175.8 (4)	O11^i^—Cd1—O9—S2	−141.33 (17)
C7—C8—C2—C3	−0.1 (6)	O1W—Cd1—O9—S2	175.0 (2)
C7—C8—C2—C1	176.9 (3)	O11^ii^—Cd1—O9—S2	−104.26 (19)
C4—C3—C2—C8	−2.8 (6)	O3—Cd1—O9—S2	−33.2 (2)
C4—C3—C2—C1	−179.9 (4)	O4—Cd1—O9—Cd2	−120.77 (11)
C15—C16—C10—C11	−1.3 (6)	O5—Cd1—O9—Cd2	−43.79 (10)
C15—C16—C10—C9	174.8 (4)	O11^i^—Cd1—O9—Cd2	71.53 (19)
O4—C9—C10—C11	0.5 (6)	O1W—Cd1—O9—Cd2	27.87 (10)
O3—C9—C10—C11	177.8 (4)	O11^ii^—Cd1—O9—Cd2	108.61 (11)
O4—C9—C10—C16	−175.7 (4)	O3—Cd1—O9—Cd2	179.70 (8)
O3—C9—C10—C16	1.6 (6)	O10—S2—O12—Cd3	−132.4 (5)
C10—C16—C15—C14	−0.5 (6)	O11—S2—O12—Cd3	106.4 (5)
O8—S1—O6—Cd2	118.20 (19)	O9—S2—O12—Cd3	−12.2 (6)
O7—S1—O6—Cd2	−118.58 (19)	N1—Cd3—O12—S2	93.6 (5)
O5—S1—O6—Cd2	−3.0 (2)	O1^iv^—Cd3—O12—S2	−48.9 (5)
O8—S1—O6—Cd3	−15.0 (2)	O3W—Cd3—O12—S2	155.9 (5)
O7—S1—O6—Cd3	108.25 (19)	O6—Cd3—O12—S2	24.8 (5)
O5—S1—O6—Cd3	−136.19 (17)	O5^v^—Cd3—O12—S2	−123.9 (5)
N3^iii^—Cd2—O6—S1	103.62 (19)	C16—C10—C11—C12	1.0 (6)
O2W—Cd2—O6—S1	−128.4 (3)	C9—C10—C11—C12	−175.1 (3)
O9—Cd2—O6—S1	−62.93 (18)	C10—C11—C12—N4	177.6 (4)
O2^iv^—Cd2—O6—S1	−161.77 (19)	C10—C11—C12—C14	1.0 (6)
O1W—Cd2—O6—S1	10.2 (2)	C16—C15—C14—C12	2.5 (6)
N3^iii^—Cd2—O6—Cd3	−120.60 (13)	C16—C15—C14—N3	−177.2 (4)
O2W—Cd2—O6—Cd3	7.4 (4)	N4—C12—C14—C15	179.8 (4)
O9—Cd2—O6—Cd3	72.85 (12)	C11—C12—C14—C15	−2.8 (6)
O2^iv^—Cd2—O6—Cd3	−25.99 (11)	N4—C12—C14—N3	−0.4 (4)
O1W—Cd2—O6—Cd3	145.96 (10)	C11—C12—C14—N3	176.9 (4)
N1—Cd3—O6—S1	−60.98 (18)	O12—S2—O11—Cd1^v^	24.1 (2)
O1^iv^—Cd3—O6—S1	−154.58 (19)	O10—S2—O11—Cd1^v^	−97.64 (18)
O12—Cd3—O6—S1	82.68 (18)	O9—S2—O11—Cd1^v^	143.85 (14)
O3W—Cd3—O6—S1	21.2 (2)	O12—S2—O11—Cd1^ii^	−171.4 (3)
O5^v^—Cd3—O6—S1	130.18 (16)	O10—S2—O11—Cd1^ii^	66.9 (3)
N1—Cd3—O6—Cd2	162.54 (14)	O9—S2—O11—Cd1^ii^	−51.6 (3)
O1^iv^—Cd3—O6—Cd2	68.94 (12)	N1—C6—N2—C5	0.1 (5)
O12—Cd3—O6—Cd2	−53.79 (12)	C4—C5—N2—C6	−176.5 (4)
O3W—Cd3—O6—Cd2	−115.24 (12)	C7—C5—N2—C6	1.4 (4)
O5^v^—Cd3—O6—Cd2	−6.3 (2)	C11—C12—N4—C13	−175.9 (4)
O8—S1—O5—Cd1	−44.7 (3)	C14—C12—N4—C13	1.1 (4)
O7—S1—O5—Cd1	−167.6 (2)	C8—C2—C1—O1	17.5 (5)
O6—S1—O5—Cd1	74.6 (3)	C3—C2—C1—O1	−165.4 (4)
O8—S1—O5—Cd3^i^	106.28 (17)	C8—C2—C1—O2	−161.8 (3)
O7—S1—O5—Cd3^i^	−16.65 (19)	C3—C2—C1—O2	15.2 (5)
O6—S1—O5—Cd3^i^	−134.47 (15)	O2—C1—O1—Cd3^iv^	7.8 (5)
O4—Cd1—O5—S1	72.8 (2)	C2—C1—O1—Cd3^iv^	−171.5 (3)
O9—Cd1—O5—S1	−42.5 (2)	C15—C14—N3—C13	179.3 (4)
O11^i^—Cd1—O5—S1	170.1 (2)	C12—C14—N3—C13	−0.5 (4)
O1W—Cd1—O5—S1	−119.2 (3)	C15—C14—N3—Cd2^vi^	19.9 (6)
O11^ii^—Cd1—O5—S1	−102.4 (3)	C12—C14—N3—Cd2^vi^	−159.9 (3)
O3—Cd1—O5—S1	73.8 (3)	C14—N3—C13—N4	1.3 (5)
O4—Cd1—O5—Cd3^i^	−74.72 (14)	Cd2^vi^—N3—C13—N4	162.0 (3)
O9—Cd1—O5—Cd3^i^	169.89 (14)	C12—N4—C13—N3	−1.5 (5)
O11^i^—Cd1—O5—Cd3^i^	22.53 (15)	O1—C1—O2—Cd2^iv^	−93.2 (4)
O1W—Cd1—O5—Cd3^i^	93.22 (14)	C2—C1—O2—Cd2^iv^	86.1 (4)
O11^ii^—Cd1—O5—Cd3^i^	110.02 (18)	O4—Cd1—O1W—Cd2	86.1 (2)
O3—Cd1—O5—Cd3^i^	−73.80 (18)	O9—Cd1—O1W—Cd2	−25.60 (9)
O3—C9—O4—Cd1	−0.9 (4)	O5—Cd1—O1W—Cd2	62.39 (10)
C10—C9—O4—Cd1	176.4 (3)	O11^i^—Cd1—O1W—Cd2	177.65 (10)
O9—Cd1—O4—C9	−101.8 (2)	O11^ii^—Cd1—O1W—Cd2	−108.66 (10)
O5—Cd1—O4—C9	179.7 (3)	O3—Cd1—O1W—Cd2	−137.43 (17)
O11^i^—Cd1—O4—C9	71.2 (3)	N3^iii^—Cd2—O1W—Cd1	−146.77 (11)
O1W—Cd1—O4—C9	157.1 (2)	O2W—Cd2—O1W—Cd1	112.47 (11)
O11^ii^—Cd1—O4—C9	−3.6 (3)	O6—Cd2—O1W—Cd1	−57.02 (12)
O3—Cd1—O4—C9	0.5 (2)	O9—Cd2—O1W—Cd1	25.96 (9)
O4—C9—O3—Cd1	0.8 (4)	O2^iv^—Cd2—O1W—Cd1	86.2 (4)
C10—C9—O3—Cd1	−176.5 (3)		

Symmetry codes: (i) *x*−1, *y*, *z*; (ii) −*x*+2, −*y*+1, −*z*+1; (iii) *x*, *y*−1, *z*; (iv) −*x*+2, −*y*, −*z*; (v) *x*+1, *y*, *z*; (vi) *x*, *y*+1, *z*.

### Hydrogen-bond geometry (Å, °)

**Table:**

*D*—H···*A*	*D*—H	H···*A*	*D*···*A*	*D*—H···*A*
N2—H2···O10^vii^	0.86	1.98	2.836 (4)	176
N4—H4A···O7^viii^	0.86	1.98	2.716 (4)	143
O1W—H1W···O3^ix^	0.85	1.91	2.736 (4)	163
O1W—H2W···O2^x^	0.85	1.91	2.734 (4)	165
O2W—H3W···O3^ii^	0.85	1.99	2.770 (4)	153
O2W—H4W···O10^ii^	0.85	2.01	2.687 (4)	136
O3W—H5W···O8^vii^	0.85	2.23	2.925 (4)	139
O3W—H6W···O4^v^	0.85	2.09	2.918 (4)	166

Symmetry codes: (vii) −*x*+2, −*y*+1, −*z*; (viii) −*x*+1, −*y*+1, −*z*; (ix) −*x*+1, −*y*+1, −*z*+1; (x) −*x*+1, −*y*, −*z*; (ii) −*x*+2, −*y*+1, −*z*+1; (v) *x*+1, *y*, *z*.
